# Computational Analysis of a Multi-Layered Skin and Cardiac Pacemaker Model Based on Neural Network Approach †

**DOI:** 10.3390/s22176359

**Published:** 2022-08-24

**Authors:** Zuzana Psenakova, Maros Smondrk, Jan Barabas, Mariana Benova, Rafał Brociek, Agata Wajda, Paweł Kowol, Salvatore Coco, Grazia Lo Sciuto

**Affiliations:** 1 Department of Electromagnetic and Biomedical Engineering, Faculty of Electrical Engineering, University of Zilina, Univerzitna 1, 01026 Zilina, Slovakia; 2 Department of Mathematics Applications and Methods for Artificial Intelligence, Faculty of Applied Mathematics, Silesian University of Technology, 44-100 Gliwice, Poland; 3 Institute of Energy and Fuel Processing Technology, 41-803 Zabrze, Poland; 4 Department of Mechatronics, Silesian University of Technology, Akademicka 10a, 44-100 Gliwice, Poland; 5 Department of Electrical, Electronics and Informatics Engineering, University of Catania, Viale Andrea Doria 6, 95125 Catania, Italy

**Keywords:** pacemaker, hypodermis layer thickness, feedforward neural network

## Abstract

The presented study discusses the possible disturbing effects of the electromagnetic field of antennas used in mobile phones or WiFi technologies on the pacemaker in the patient’s body. This study aims to obtain information on how the thickness of skin layers (such as the thickness of the hypodermis) can affect the activity of a pacemaker exposed to a high-frequency electromagnetic field. This study describes the computational mathematical analysis and modeling of the heart pacemaker inserted under the skin exposed to various electromagnetic field sources, such as a PIFA antenna and a tuned dipole antenna. The finite integration technique (FIT) for a pacemaker model was implemented within the commercially available CST Microwave simulation software studio. Likewise, the equations that describe the mathematical relationship between the subcutaneous layer thickness and electric field according to different exposures of a tuned dipole and a PIFA antenna are used and applied for training a neural network. The main output of this study is the creation of a mathematical model and a multilayer feedforward neural network, which can show the dependence of the thickness of the hypodermis on the size of the electromagnetic field, from the simulated data from CST Studio.

## 1. Introduction

**Figure 1:**
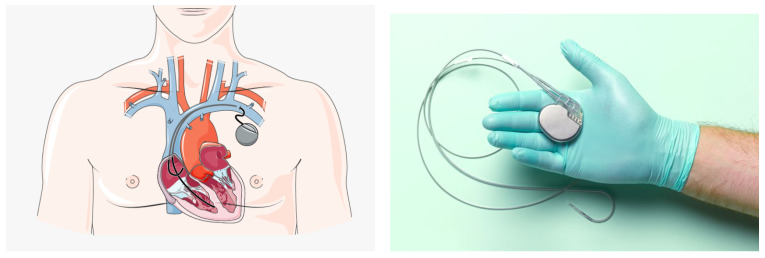
Cardiac Pacemaker [1].

Approximately one million patients worldwide receive a cardiac pacemaker, considered an implantable medical device able to detect the electrical activity of the heart and stimulate it when the heart is inadequate (Figure 1). The device can come into daily contact with electronic devices and electromagnetic fields (cell phones, electric razors, microwave ovens, high-speed trains, metal detectors, etc). The most intense interference can occur with receiver–transmitter radio systems and most commonly with the phone.

The probability of interference is high during the activation of the call signal and, in some cases, 3 or 5 s before. As the distance from the device between the cardiac pacemaker (PM) and the mobile phone increases to a few centimeters, the amount of interference that the electronic system produces at radio frequency (RF) is significantly reduced. The past studies reported an incidence of an electromagnetic interference (EMI) variable of 20–30% between cardiac pacemakers (PM) and cellular phones. The latest studies reported a reduction in the electromagnetic interference at about 1–5% [2]. The previous PMs had a higher incidence of interference; however, the new generation of PMs are equipped with RF filters built into the internal circuit. Few studies have investigated the mechanism by which electromagnetic fields interfere with the PM. However, they show that the susceptibility of the PM to electromagnetic fields depends on the architecture of the device’s internal circuit [3,4]. EMI can affect a pacemaker to deliver irregular pulses that regulate the heart’s rhythm, or it can ignore the heart’s natural rhythm given pulses at a fixed rate [5,6,7]. The RF transmitted from the mobile phone can be guided into the PM via the catheter, and the internal conductor of the catheter can act as an antenna. In particular, the PM’s outer casing is made of titanium, forming a barrier against body fluids and the electromagnetic fields. Demodulation can occur if the filter does not provide suitable closure to the RF. The RF signal is demodulated at low frequencies, reaches the PM’s internal circuits, and it is interpreted as the electrical activity of the heart and can interfere with the PM’s operation. It is very important to recognize that these interferences can cause clinically significant consequences (i.e., the symptoms experienced by the patients) only if the patient was totally dependent on the PM. In order to prevent interference, it is pertinent to bring the levels of static magnetic B-fields to 0.5 mT as recommended by the ICNIRP, and for the American Conference of Governmental Industrial Hygienists, electric fields at 50 Hz are 1 kV/m and magnetic fields are 100 mT [8,9]. The potential of electromagnetic interference can be effectively reduced by practical information, accurate information for workers, and appropriate warning signs near to the electromagnetic radiation system at the pacemaker interference level. The precautionary measures provide alternative solutions for pacemakers in proximity systems (metal detectors and anti-theft devices) or prohibit access to pacemakers from EMF sources. Lee et al. [10] have evaluated the magnetic field strengths of eight different models of portable headphones to determine if they can cause clinically relevant magnetic disorders in 100 patients. Many studies have assessed the prevalence of interference and the potential for serious clinical risk resulting from the exposure of permanently implanted pacemakers to mobile phones and the miniaturized implantable antenna integrated with systems operating at 2.4 GHz for monitoring cardiac activities [11,12]. Cardiac pacemakers are powered by nanogenerators through in vivo energy generation to generate stimulation pulses [13]. Pulsating electrical signals are generated by microcontrollers, and the conversion and excitation mechanism that emulate the behavior of artificial heart pacemakers are studied in [14]. In daily life, patients with a PM are potentially vulnerable to the adverse effects of many EMI sources [15]. That electromagnetic fields have a great effect on the human body is well known. In several papers, the whole pacemaker–heart system has been explored using multiple analysis tools and models [16,17,18]. In [16], an integrated simulation of pacemaker models and heart models are developed in the Prototype Verification System (PVS) to gain additional confidence that the software design complies with specific safety requirements. A hybrid heart model in Simulink is formulated at the level of cardiac cells and can be adapted to patient data and incorporates stochasticity [19]. Electromagnetic fields are presented everywhere in domestic and working environments and in public spaces, and they are likely to interfere with relatively complex electronic active medical implants also at a low frequency in electromagnetic fields (0–100 kHz). The risk of interference (EMI) is actually complex to analyze, because it is not exclusively dependent on the intensity of the EM field and the duration of exposure but also factors specific to the type of implant which give it variable sensitivity (a device without the probe or with the probe implanted, measurement or stimulation, and uni- or bipolar) and its programming parameters, as well as its location in the wearer’s body and morphology [20,21,22]. EMI signals in the 10 to 60 Hz frequency range can negatively affect cardiac devices because they overlap the cardiac signal range. This is because the amplitude and frequency of human muscle potentials overlap the same range as the cardiac signals [20,23,24]. However, in this study, a relationship based on an artificial neural network (ANN) has been proposed between the electric field distribution and the cardiac pacemaker placed under the multilayer structure, characterized by various subcutaneous tissues of the skin exposed to 2.4 GHz RF with a power of 0.5 W in the air. The proposed neural network is a multilayer feedforward neural network composed of an input layer, an output layer, and two hidden layers. In our case, the cardiac pacemaker has been exposed to radiated fields by antennas, evaluating the effects for different categories of the human body, such as underweight, normal or healthy weight, overweight, and obesity. The paper is organized as follows: Section 2 introduces the cardiac pacemaker implantation. Section 3 describes the numerical model of the multilayered tissue, including the dipole antenna and the planar inverted-F-type antenna (PIFA). Section 4 describes the developed neural network. Section 5 discusses the results obtained by the ANN. Section 6 closes with the conclusions.

## 2. Pacemaker Implantation

The heart activity depends on the continuous and physiological functioning of the sinoatrial node cells that act as the natural pacemakers. In pathological situations, the natural pacemaker ceases to operate or becomes unreliable, or if the stimulation impulse is not able to reach the cardiac muscle due to a block in the conduction system, the heart’s pumping action is impaired. In these situations, a suitable electrical stimulation can be required to regulate the heart rhythms. This electrostimulation is provided by the pacemaker. The cardiac pacing mode allows to distinguish two types of pacemakers: implantable and external. The external pacemakers are designed to return the normal heart rhythm in case of cardiac arrest, in situations where it is required for a short period of time. Frequently, the external pacemaker is used to correct temporary conduction disturbances due to cardiac surgery [1]. When the patient recovers and restores the normal heartbeat, the use of the pacemaker will stop. The internal pacemakers are used in the case of prolonged stimulation. This is the cause of permanent damage that prevents the normal auto-activation of the heart. The implantable cardiac pacemaker can be implanted completely under the skin. The application of a pacemaker requires a surgical intervention, the most important part of which consists of connecting the pacemaker to the heart.

Although pacemaker implantation is considered to be low risk, problems and technical failures such as pneumothorax and lead dislodgement, superficial phlebitis, and hematomas might occur during the surgery [25,26].

This procedure is performed under local anesthesia, using the cardiac catheterization techniques, with the electrodes placed on the right hemi-thorax and a pacemaker wire inserted into a vein. The wire is connected to the pacemaker [27,28,29]. The pacemaker is then inserted under the skin through a small skin incision to permanently secure it. The skin is a fairly complex organ, consisting of three main layers: the epidermis, the dermis, and the hypodermis, each of which has a specific function. The epidermis is the most superficial layer of the skin, and it is composed of various layers that represent a dynamic process. In fact, the epidermis is constantly renewed. Younger cells are formed and replace the old cells. The latter die and are removed in a transformation process that takes about three weeks. The main function of the skin is to protect the body from external aggression. The dermis, which lies below the epidermis, is formed, primarily, by fibrous connective tissue, where the collagen, elastic, and reticular fibers have not only the direction parallel to the skin surface but also perpendicular, providing elasticity to the skin and extensibility within certain limits. The dermis contains special structures, such as a complex system of blood vessels, nerves, and sensory nerve receptors that collect tactile stimuli, such as heat, cold, rough, smooth, pressure, pain, etc., exerted on the skin and transmit them to the brain, the sweat glands, and the sebaceous glands [30,31]. The hypodermis or subcutaneous tissue is the deepest layer of the skin and is mostly composed of connective tissue that protects the body from the cold and acts as energy reserves. Skin thickness varies widely in different parts of the body (0.04 mm in the eyelids up to 4 mm in the feet), even in lean and obese people. In the introduced paper, we create a multilayered model of skin tissue for an investigation of how thick the fat tissue (hypodermis) or other layers of the skin must be for the use of a pacemaker to be safe for the patient. Because we found out in previous works that it is mainly the thickness of the hypodermis that is decisive for the depth of the EMF penetration into the tissue, we develop the multilayered skin with the pacemaker model and utilize the results of the neural network training. The trained neural network is subsequently used for the prediction of electric field strength in deeper layers of exposed tissue and the probable outcomes of exposure to the implanted pacemaker.

## 3. Numerical Modeling of Electromagnetic Field (EMF)

In recent years, the use of wireless technologies has become very widespread. This has resulted in an increase in the intensity and spatial presence of electromagnetic fields. This is particularly important for pacemaker (PM) implantation patients, who may thus be exposed to excessive radiation. The most commonly used devices include cell phones, Bluetooth, or WiFi devices. They typically operate in the 2.4 GHz center frequency band, which is an unlicensed ICM band. The exact effect of radiation exposure on the human body has not yet been established completely. This is still the subject of research. Hence, the presence of some metallic implants in the superficial layers of tissue (panniculus) can assume antenna functions. In this connection, a possible local increase in the electromagnetic field of the human body by PM is recognized. This occurs in terms of electric field strength or specific absorption rate (SAR) values. This parameter is a dosimetric quantity. It indicates the rate of energy absorption by human tissue during exposure to RF electromagnetic radiation. The local distribution of the electromagnetic field is significantly influenced by the dielectric properties, as well as the size of the metal implant, the thickness of the surrounding tissue, or the shape and orientation relative to the field source [32,33]. Therefore, the electric field distribution within a multilayer structure (skin tissue and PM model) that was irradiated by an external RF-EMF source was analyzed in this study. The result of the research was the calculation of the electric field distribution ($\vec{E}$ [V.m^−1^]) given by the following equation:

(1) $$▿\times \frac{1}{\mu_{r}}\left(▿\times \vec{E}\right)-k_{0}^{2}\left(\varepsilon_{r}-\frac{j\sigma }{\omega \varepsilon_{0}}\right)\vec{E}=0$$

where $k_{0}$—wave vector in free space (m^−1^), ▿—rotation vector operator, $\varepsilon_{r}$—relative permittivity, $\varepsilon_{0}$—vacuum permittivity (F·m^−1^), $\mu_{r}$—relative permeability, σ—electrical conductivity (S·m^−1^), ω—angular wave frequency (rad·s^−1^), and *j*—imaginary unit [34].

To achieve the computing electric field distributions, the application of the finite integration technique (FIT) was chosen. In the study, the commercial simulation software, CST Microwave Studio, was used. The hexahedral mesh was applied in the time-domain solver based on the FIT.

### 3.1. Electromagnetic Field Source

Two sources of RF EMF were used—a dipole antenna and a planar inverted-F-type antenna (PIFA). A generalized tuned dipole antenna model was used because it is easy to model for specific resonant frequencies, but it is rarely used in aforementioned technologies.

**Figure 2:**
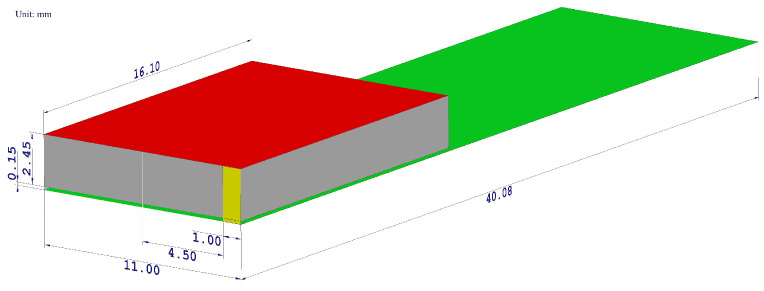
The geometrical layout of a designed PIFA antenna model. The radiating element is highlighted by the red color, the ground plane by the green color, the shorting plate by the yellow color, the feeding pin by the blue color, and the air gap by the gray color. All dimensions are shown in millimeters.

It is modeled as a long cylinder fed by a discrete port situated in the center of the longitudinal cylinder axis with a reference impedance of 50 Ω (Figure 2).

**Figure 3:**
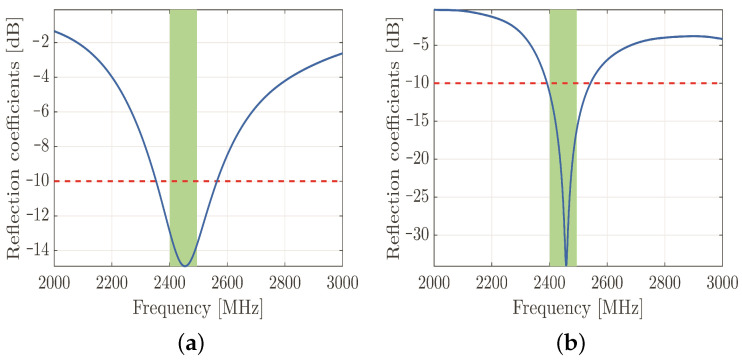
Simulated reflection coefficients of designed dipole antenna. The reflection coefficients are plotted along the vertical axis with the blue line, the threshold value of −10 dB is represented by the red dashed line, and the green rectangle represents the 2.45 GHz frequency band of IEEE 802.11b standard (**a**). Simulated reflection coefficients of designed PIFA antenna. The reflection coefficients are plotted along the vertical axis with the blue line, the threshold value of −10 dB is represented by the red dashed line, and the green rectangle represents the 2.45 GHz frequency band of IEEE 802.11b standard (**b**).

The cylinder was modeled as a lossy copper metal. The geometric properties of the dipole antenna are summarized in Table 1. The frequency response of the simulated reflection coefficients is shown in Figure 3a to confirm that the designed antenna resonates in the desired frequency band.

On the other hand, the currently prevalent antenna for hand-held devices is the PIFA—due to both its compactness and size. This device is a quarter-wave monopole antenna. It is constructed by two parallel conducting plates, which are connected to the ground through a shorting pin. The resonant frequency of the PIFA depends primarily on the width of the shorting pin and the dimensions of the radiating element. After several parametric simulations, the optimal geometrical parameters have been obtained by maintaining the appropriate reflection coefficients in the desired frequency band (Figure 3b). The material of both the radiating element and ground plane was copper, and the aforementioned elements were separated by an air gap. The radiating element was fed by a discrete port with a reference impedance of 50 Ω.

The PIFA, namely, includes a linear inverted-F antenna (IFA) with the wire radiator element of IFA replaced by a plate in order to improve the performance in terms of bandwidth, suitable to be used in portable wireless devices, especially in mobile handsets. For the mobile devices market, several advantages are offered by it, guaranteeing low profile and manufacturing costs, small size, easy fabrication, simple structure compared to other microstrip antennas, allowing to locate it in structure such as the back cover of mobile terminal, low SAR value (specific absorption rate), and small backward radiation toward the user’s head, reducing the signal absorption and increasing the antenna performance. Typically, the inverted-F antenna (IFA) consists of a rectangular planar element positioned on ground plane, a short circuiting plate or pin, and a feeding mechanism for the planar element. It is an alternative to the monopole antenna, able to reduce the antenna height maintaining the resonant frequency. In the inverted-F antenna, the top section is folded down so as to be parallel with the ground plane, which introduces a capacitance to the input impedance that is compensated by using a short-circuit stub connected to the ground plane. The current intensity in the IFA causes excitation of currents in the ground plane, then the resulting electromagnetic field is produced by the interaction of two contributions: IFA and an image of itself in the ground plane. When the ground-plane size is infinite or much larger than IFA, it acts like a perfect energy reflector.

**Figure 4:**
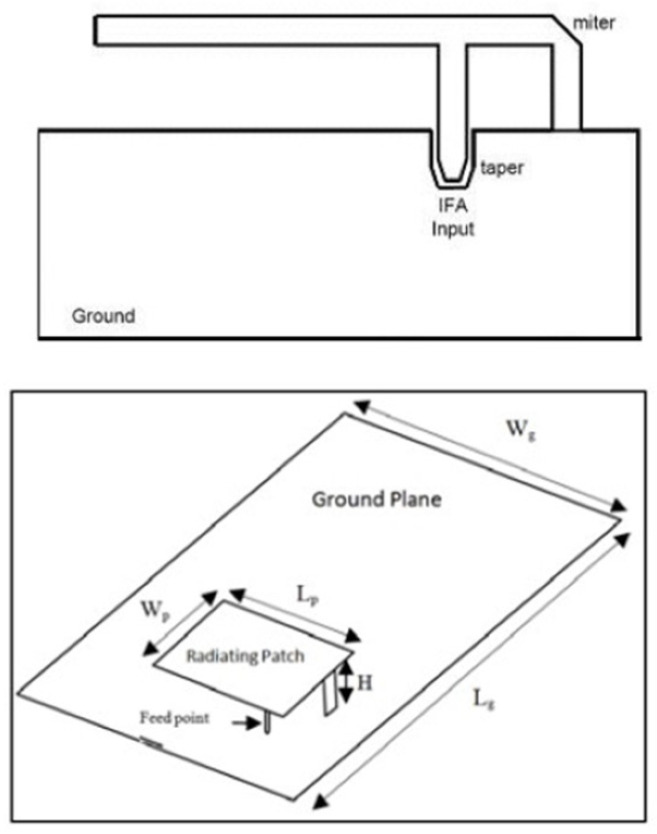
Inverted-F antenna above and architecture PIFA structure below [35,37].

More specifically, the required PCB (Printed Circuit Board) ground-plane length is about one-quarter of the operative wavelength (λ/4): if the ground plane is too much longer than (λ/4), the radiation pattern gradually becomes multilobed, but on the other hand, if it is too much smaller than (λ/4), the tuning becomes very difficult and the performance decreases. In order to obtain an omnidirectional far-field pattern and a 50 Ω impedance, IFA has to be located close to the edge of the PCB, as shown in Figure 4. As shown in Figure 4, the miter avoids a straight angle in the microstrip bend, which can cause a low stub current intensity. The taper, on the other hand, compensates for the step transition between the antenna and the microstrip feed. Then, the planar inverted-F antenna (PIFA) has a self-resonating structure that presents a totally resistive load impedance at the operation frequency, with a wide bandwidth and high efficiency. Variation of length, distance, and location of the feed; shorting point; and height of the radiator affect the electrical performance of these antenna structures. As well as the advantages set out above, PIFA provides moderate to high gain in vertical and horizontal polarization, a very useful option, especially for cases where the antenna orientation is not static and the reflections come from different corners of the environment; in those cases, the total field is the vector sum of horizontal and vertical polarization. Because the major drawback of PIFA is the narrow bandwidth, for mobile phones and other handheld devices, the bandwidth needs to be extended, for instance, by raising the height of the shorting plane, i.e., increasing the volume, or modifying the ground-plane size [35].

Typical configuration of PIFA is shown in Figure 4. The antenna is fed through feeding pin which connects to the ground plane through the dielectric substrate. The shorting pin and shorting plate ensure good impedance matching achieved with the patch above ground plane of size less than (λ/4). Resulting PIFA structure is more compact in size than conventional (λ/2) patch antennas. The resonant frequency can be calculated by using the following Equation (2) [36]:

(2) $$f_{0}=\frac{c}{4(W_{p}+L_{p})}$$

where:

- *c* is the speed of the light;
- $W_{p}$ is the width of the top plate of PIFA;
- $L_{p}$ is the length of the top plate of PIFA;
- $f_{0}$ is the resonant frequency.

The equation referred in Equation (3) provides an inaccurate approximation not considering all parameters that can affect the resonant frequency, such as the width of the shorting plate: the reduction in shorting plate width involves a lower resonant frequency and vice versa. A more accurate equation to design the antenna in practice, calculating the resonant frequency, is provided in [38,39]:

(3) $$f_{0}=\frac{c}{4(L_{p}+\Delta_{l}+k_{1}{\left(W_{p}-W_{s}\right)}^{2}+k_{2}\left(W_{p}-W_{s}\right))}$$

where:

- $W_{s}$ is the width of the shorting plate;
- $\Delta_{l}=2.741$;
- $k_{1}=0.0188$;
- $k_{2}=0.0767$.

### 3.2. Multilayered Tissue Model

**Figure 5:**
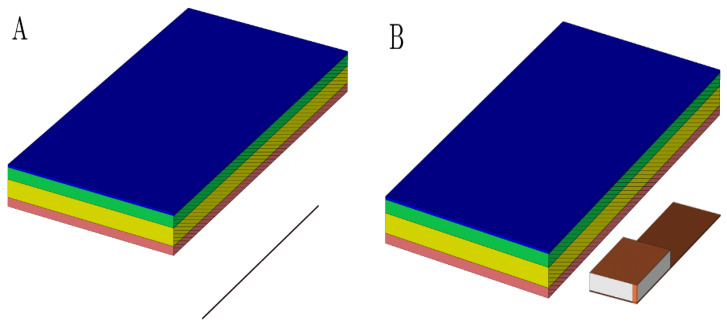
The geometrical layout of a used multilayered model with a dipole antenna (**A**) and PIFA antenna (**B**) model. The perspective view with vertical cutting plane crossing the longitudinal axis of the antenna was used to emphasize the mutual distance between the antenna and dermis. It was set up to 10 mm. Pink colored block is an epidermis layer, yellow block is a dermis layer, green block is a hypodermis layer, and blue block is a pacemaker layer.

In order to conduct the study, a tissue model was established including skin tissue and PM. The layers of epidermis, dermis, and PM casing are maintained of constant thickness, and the thickness of the hypodermis was varied. The database [40] was used to obtain the dielectric properties of skin layers. The given data are summarized in Table 2. Titanium was chosen for the PM casing material. In all scenarios, the antenna was placed 10 mm away from the epidermis layer (Figure 5).

Simulations were performed on four different hypodermis tissue layer thicknesses. The results of the calculated field intensity distribution within the multilayer structure irradiated by the tuned dipole antenna are discussed in [41], and the PIFA antenna model results are detailed in [42].

In general, the numerical simulation of models lacking symmetrical axes or models with complex geometrical designs are time consuming and require considerable computational resources. Therefore, the aim of this contribution is to investigate whether neural networks can overcome these limitations in terms of electric field strength calculation.

## 4. Neural Network Basic

Modern computing and numerical modeling are used to examine the interference between the magnetic fields within the body [43]. Artificial neural networks (ANNs) are biologically inspired by computational neural systems [44,45]. The ANN models have the architecture inspired by a biological nervous system, consisting of processing elements that are connected to other processing elements. Typically, the neurons are connected to each other by weighted links and arranged in a layer or vector, with the output of one layer serving as the input to the next layer and possibly other layers. All the processes in ANN models, such as data collection and analysis, are computed through the learning and training methods for the clustering, classification, and simulation. There is a wide variety of neural network models, such as the Restricted Boltzmann Machine (RBM), convolutional neural networks (CNN), and recurrent neural networks (RNN). However, the CNNs are useful for image processing and computer vision, as well as recurrent neural networks, deep networks, and deep belief systems. In this case, the multilayer neural network with appropriate weights can approximate the complicated input–output function for modeling and forecasting. By using the data obtained from the simulations, a simple neural feedforward network is trained in order to obtain a simplified relation between the hypodermis layer thickness and the attenuation of the electromagnetic field in the skin tissues. The selected neural network is a multilayer feedforward neural network composed of the input layer, the output layer, and hidden layers. For the training, a fast Levenberg–Marquardt algorithm, the well-known gradient descent with adaptive learning rate backpropagation algorithm, and the relative learning parameters are considered. Thus, the learning rules update the weights and bias levels of a network when a network simulates a specific data environment. To prevent overfitting in the networks, the early stopping rule is used. The method used during the implementation of the neural network was to separate a set of available data into three subsets: the training, validation, and testing sets. The training set is used as the primary set of data that is provided as the input to the neural network for learning and adaptation. The inputs to the neural network are the subcutaneous layer thickness, and the targets are the electric field strengths for different exposures of the tuned dipole and PIFA antennas. In this work, the relationship between the subcutaneous layer thickness and the electric field strength for different exposures of the tuned dipole and PIFA antennas is established using a neural network approach.

**Figure 6:**
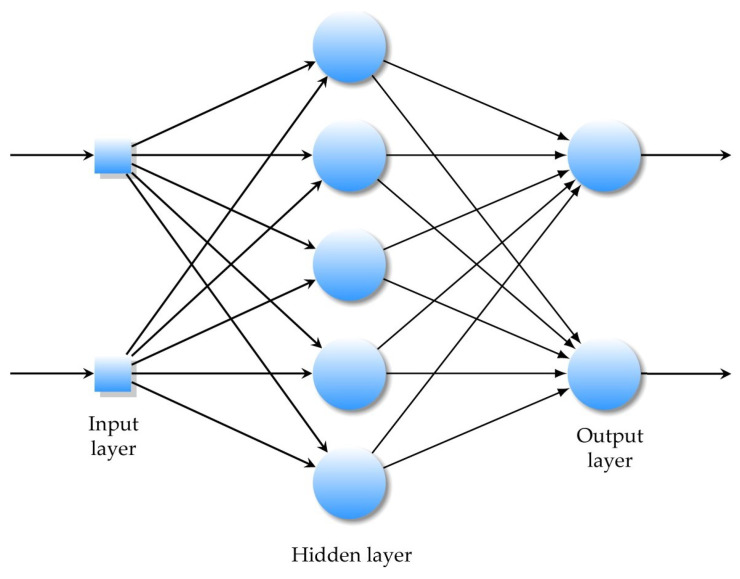
A multilayer feedforward ANN with one hidden layer.

The neural network input/output transfer functions can be easily obtained through a supervised learning process based on empirical data, Figure 6.

The network is trained by the appropriate algorithms, usually backpropagation learning algorithms. The latter is used to change, in the same network, the weights $w_{i}$ and parameters (threshold values) to minimize the sum of the squared error function.

The weight of an input is the number which, when multiplied with the input $x_{i}$, gives the weighted input. The function *g* is the unit’s activation function.

(4) $$y\left(x\right)=g\left(\sum_{i=1}^{d}w_{i}x_{i}+w_{0}\right)$$

where

(5) $$w_{1}=iw\left[1,1\right],\;w_{2}=iw\left[2,1\right],\;b_{1}=b\left[1\right],\;b_{2}=b\left[2\right]$$

Each input weight, layer weight, and bias vector has as many rows as the size of the *i*-th layer, after the training network bias and weights change. In our case, iw[1,1] is the weight to layer 1 from input 1, lw[2,1] is the weight to the layer, b[1] is the bias to layer 1, and b[2] is the bias to layer 2.

In a multilayer feedforward neural network, artificial neurons are arranged in layers, and all the neurons in each layer are connected to all the neurons in the next layer.

Each connection between these artificial neurons is assigned a weight value that represents the weight of the connection.

## 5. Analysis of the Results

The multilayer feedforward neural network computes the relationship between the subcutaneous layer thickness and the electric field intensity for different exposures of the tuned dipole and the PIFA antennas. The inputs to the neural network are the subcutaneous layer thickness, and the targets are the electric field strengths for different exposures of the tuned dipole and PIFA antennas. In this work, the relationship between the subcutaneous layer thickness and the electric field strength for different exposures of the tuned dipole and PIFA antennas is established using a neural network approach.

In the feedforward process, the external input values are first multiplied by their weights and summed. The output y=f(x) is a weighted sum function, called the activation function. The relationship between the input variable *x* and the output variable *y* is achieved by adjusting the parameters and weights to reduce errors. The process of finding a set of weights so that the network produces the desired output for a given input is called training. Neural networks learn the relationships between different input and output patterns. The feedforward backpropagation neural network was used to determine the nonlinear mapping from the input vector of the tissue layers, specifically four different subcutaneous layer thicknesses, and the electric field strength using the planar inverted-F-type antenna (PIFA) and the tuned dipole antenna.

The input is defined by the thickness of the hypodermis tissue in terms of the BMI classes: 2 mm for underweight, 5 mm for normal weight, 10 mm for overweight, and 18 mm for obese. The output is expressed by the electric field strength obtained by the tuned dipole antenna. The feedforward backpropagation neural network is composed by an input layer with one neuron arranged in the output layer, using a linear transfer function, and one hidden layer with a hyperbolic tangent sigmoid transfer function.

The network training function (trainbfg) is used to update the weight and bias values according to the BFGS quasi-Newton method.

**Figure 7:**
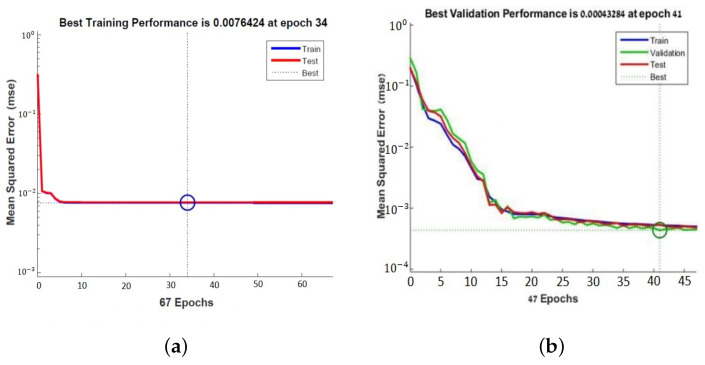
Learning curves of the neural network for PFA antenna (**a**) and for tuned antenna (**b**).

The learning curve plots the optimal value of the obtained model for the training and validation datasets. The MSE measures the average of the squares of the errors between the desired signal and the primary signal input. Learning curves can be used to diagnose whether the training or validation datasets are not relatively representative of the problem domain (underfit, overfit, or well-fit model). The learning curves, shown in Figure 7, pointed out the good performance of the ANN, and after 41 epochs, a mean squared error (MSE) of 0.00043284 and an excellent generalization, due to the fact that the test curve is always under the training curve, are obtained, as shown in Figure 7a. By Equation (4), *x* is the input value of the subcutaneous tissue thickness array and *y* is the output absolute value of electric field strength array.

(6) $$y\left(x\right)=\frac{10.7634}{1+exp(-2\times (-1.1992\times x-1.968))}-0.94$$

The same neural network is used with a network training function trainbr that updates the weight and bias values according to the Levenberg–Marquardt optimization. Iteratively, the optimization technique compares numerous solutions until a satisfactory one is identified.

It minimizes the combination of the root mean squared errors and weights, and then determines the appropriate combination, so as to produce a well-generalized network. This process is called Bayesian regularization. The learning curve showed the performance of the second neural network obtained after 34 epochs. The MSE is 0.0076424 which shows a good generalization (Figure 7a).

In the second case, the relationship between the skin layer thickness input values (x) and the electric field strength distribution (y) of the PIFA antenna proposed as the source of near-field electromagnetic exposure is presented.

The same neural network is used with trainbr. The latter is a network training function that updates the weight and bias values according to the Levenberg–Marquardt optimization. It minimizes the combination of root mean squared errors and weights, determines the appropriate combination, and generates a well-generalized network. This process is called Bayesian regularization. The learning curve in Figure 7b shows the performance of the second neural network obtained after 34 epochs with an MSE of 0.0076424. The learning curves in the plot of Figure 7a,b show a good fit of the learning algorithm identified by a training and validation loss that decreases to a point of stability with a minimal gap between the two final loss values.

(7) $$y\left(x\right)=\frac{0.259}{1+exp(-2\times (-3.8274\times x-2.5193))}-0.994$$

## 6. Conclusions

The induced electromagnetic fields can cause interference with ICDs and implanted pacemakers within the body. This study describes a computational mathematical analysis and the modeling of a pacemaker implemented under the skin exposed to different electromagnetic field sources, such as a PIFA antenna and a tuned dipole antenna. The simulation results show the calculated field intensity distribution within the multilayer structure irradiated by the tuned dipole and PIFA antennas performed on four different hypodermis tissue layer thicknesses. In order to accomplish this, the finite integration technique (FIT) implemented within a commercially available simulation software, CST Microwave Studio, is used. Similarly, the equations that describe the mathematical relation between the hypodermis layer thickness and electric field, according to different expositions of the tuned dipole and PIFA antennas, are obtained by training the neural network. In particular, a relationship based on an artificial neural network (ANN) has been proposed between the electric field distribution and the cardiac pacemaker placed under the multilayer structure, characterized by various subcutaneous tissues of the skin exposed to 2.4 GHz RF with a power of 0.5 W in the air. The multilayer feedforward neural network with appropriate weights has approximated the complicated input–output function between the hypodermis layer thickness and the attenuation of the electromagnetic field in the skin tissues. The developed MLP neural network has been useful for modeling the cardiac pacemaker exposed to EMI. In future work, the ANN can be useful to predict the electromagnetic interference with the implantable cardiac pacemaker.

## Figures and Tables

**Table 1:** Geometric properties of dipole antenna model.

Parameter	Value	Unit
Center Frequency	2450	MHz
Speed of Light	299,792,458	m·s^−1^
λ	Speed of Light/Center Frequency	mm
Cylinder Length	0.46·λ	mm
Cylinder Radius	0.001·λ	mm

**Table 2:** Thickness and dielectric properties of the multilayer model (frequency of 2.4 GHz) [40].

Layer	Thickness (mm)	$\varepsilon_{r}$	σ (S·m^−1^)
Epidermis	1.5	38.1	1.44
Dermis	3	25.2	5.76
Hypodermis	2/4/10/18	30.5	3.6
Pacemaker casing	0.5	50	0.267 × 10^−6^
